# Predicting the potential distribution of three medicinal *Gentiana* species in China under climate change scenarios with the MaxEnt model

**DOI:** 10.3389/fpls.2025.1729969

**Published:** 2026-01-27

**Authors:** Jun Luo, Xinyu Li, Ying Liu, Shiyu Zhang, Anli Liu, Ying Liu, Ying Zhou

**Affiliations:** 1 Guizhou University of Traditional Chinese Medicine, Guiyang, China; 2 The First Affiliated Hospital of Guizhou University of Traditional Chinese Medicine, Guiyang, China; 3 Guizhou Key Laboratory of Modern Traditional Chinese Medicine Creation, Guiyang, China

**Keywords:** climate change scenarios, *Gentiana* species, range shift, species distribution modeling, suitable habitat

## Abstract

The genus *Gentiana* is concentrated in the Qinghai-Tibet Plateau and adjacent Hengduan Mountains, with its distribution pattern reflecting the synergistic effects of geological and climatic changes. This study employs the MaxEnt model integrated with ArcGIS spatial analysis to predict the potential geographical distribution of three medicinal Gentiana species (*G. rhodantha*, *G. cephalantha*, and *G. rigescens*) in China under current and future climate scenarios (SSP126 and SSP585). Under future climate warming, our projections indicate an overall reduction in suitable habitat area for all three species, with *G. rigescens* experiencing the most severe habitat loss. Furthermore, the centroid of suitable habitats is projected to shift towards higher latitudes and elevations, reflecting a spatial adaptation strategy to climate change. The key environmental drivers of distribution were identified: annual precipitation (Bio12) and minimum temperature of the coldest month (Bio6) primarily determine the distribution of *G. rhodantha*, while temperature seasonality (Bio4) and altitude are the dominant factors for *G. cephalantha* and *G. rigescens*. Our projections indicate an overall reduction in suitable habitat area for all three species under climate warming, with G. rigescens experiencing the most severe loss. Furthermore, the centroid of suitable habitats is projected to shift northwestward and upward in elevation. These findings highlight species-specific responses to climatic factors and provide a scientific basis for prioritizing the conservation of current highly suitable areas (e.g., Yunnan, Sichuan, and Guizhou), establishing ecological corridors, and implementing *ex-situ* conservation and sustainable cultivation practices to mitigate the impacts of climate change on these valuable medicinal resources.

## 1 Introduction

As one of the core drivers of global change, climate change exerts a profound and undeniable impact on species distribution patterns (Howarth and Viner, 2022). In recent years, with the continuous global warming, the geographical distributions of species have undergone extensive and profound restructuring (Jorda et al., 2020; Kellner et al., 2023). This process primarily disrupts key biological processes such as reproductive phenology, growth and development cycles, and population dynamics (Root et al., 2003; Memmott et al., 2007), exacerbating the loss of biodiversity and germplasm resources, which in turn triggers severe ecological crises (Thomas et al., 2004; Pecl et al., 2017). Meanwhile, habitat fragmentation caused by rapid urbanization further amplifies the negative effects of climate change, posing greater challenges to the stability and continuity of natural ecosystems. According to the IPCC Sixth Assessment Report, the global average temperature may rise by 0.3˜1.7°C under low-carbon scenarios and 2.6˜4.8°C under high-emission scenarios by 2100, with more significant seasonal changes in climate variability (Hending et al., 2022). Against this backdrop, the evolution of ecological environments within a multiscale spatiotemporal framework has become a key factor shaping species distribution patterns and the multidimensional structure of ecosystems (Liang et al., 2025). Therefore, systematically assessing the potential impacts of climate change on plant species and accurately predicting the distribution of their suitable habitats not only constitutes a core topic in basic ecology and biogeography research but also provides a scientific basis for formulating biodiversity conservation and climate adaptation strategies (Bellard et al., 2012).

The genus *Gentiana* (Gentianaceae) comprises approximately 400 species, mostly annual or perennial herbs, widely distributed in Europe, Asia, and northern Australia (Editorial Committee of Flora of China, 1988). China, as one of the centers of diversity for this genus, harbors 247 species, which are mainly concentrated in alpine regions from southwestern to northwestern China, including northwestern Yunnan, Sichuan, Tibet, Qinghai, and Guizhou provinces. These species are commonly found in habitats such as alpine screes, meadows, and thickets (Zhengyi, 1987). *Gentiana* plants are renowned for their rich secondary metabolites, including iridoids, secoiridoids, and their derivatives. Among these, gentiopicroside and swertiamarin are the main bioactive components, endowing these plants with traditional medicinal values such as clearing heat and draining dampness, and purging the heat in liver and gallbladder (Commission, 2025). Modern pharmacological studies have further confirmed their various biological activities, including hepatoprotective, anti-inflammatory, antipyretic, and antiviral effects (Cui et al., 2005; Chen et al., 2008; Li et al., 2015). However, while existing studies have mostly focused on the chemical composition and medicinal potential of *Gentiana* species, systematic investigations into their spatial distribution patterns in China and their responses to environmental factors remain insufficient. Given that climate change continues to alter the structure and function of terrestrial ecosystems, potentially leading to significant shifts and fragmentation of the suitable habitats of *Gentiana* species. Scientifically predicting changes in their potential distributions holds important practical significance for formulating effective conservation strategies and achieving the sustainable utilization of resources.

Currently, ecological niche models have become important tools for predicting the potential geographical distributions of species. Among them, various algorithms such as the Bioclimatic Model (Bioclim), Genetic Algorithm for Rule-Set Prediction (GARP), and Maximum Entropy Model (MaxEnt) have been widely applied. As one of the most representative models in current species distribution modeling, MaxEnt is favored in numerous studies due to its ability to handle incomplete data and multi-source environmental variables. By solving for the state parameters of the species-environment interaction system when entropy is maximized, MaxEnt reveals the steady-state relationship between species and the environment, thereby accurately estimating the potential suitable habitats of species (Cory et al., 2013). This model not only has a wide range of applications but also exhibits robust predictive performance (Phillips et al., 2006). Notably, the integrated application of the Maximum Entropy Model (MaxEnt) and Geographic Information System (ArcGIS) has gradually developed into a core technical framework for simulating species’ suitable habitats. This methodology has demonstrated significant value in multiple fields, including but not limited to the assessment of suitable habitats and formulation of conservation strategies for endangered flora and fauna (Ye et al., 2021a), the effective management of the spread risks of invasive species (Zhang et al., 2023), the evaluation of habitat suitability for introduction and cultivation (Liu et al., 2021), and the analysis of the spatial structure and dynamic patterns of ecosystems (Yao et al., 2025).

In this study, we integrated the aforementioned technical system and combined the spatial analysis and visualization functions of ArcGIS to conduct a comparative analysis of the current and potential distributions of suitable habitats and ecological characteristics of *Gentiana* species. The objectives of this study were: (1) to identify the dominant environmental variables shaping the current distribution of three selected Gentiana species; (2) to project their potential distribution shifts under future climate change scenarios (SSP126 and SSP585) for the periods 2041–2060, 2061–2080, and 2081–2100; and (3) to assess the implications of these changes for conservation planning and sustainable resource management of these medicinal plants.

## 2 Materials and methods

### 2.1 Species selection criteria

Among the 247 *Gentiana* species documented in China, *G. rhodantha*, *G. cephalantha*, and *G. rigescens* were selected as focal species based on the following four criteria: (1) Ecological Gradient Representation: The three species collectively span the major distributional and elevational gradients of the genus in China. *G. rhodantha* primarily occupies mid−to−low elevation humid areas, *G. cephalantha* is typical of mid− to high−elevation transitional zones, and *G. rigescens* is restricted to high−altitude regions of the Yunnan−Guizhou Plateau. Together they reflect the ecological niche differentiation of *Gentiana* from southeast to southwest and from low to high elevations. (2) Medicinal and Economic Importance: All three species are listed in the 2025 edition of the Pharmacopoeia of the People’s Republic of China or are widely used in local medicinal practices, with demonstrated hepatoprotective, anti−inflammatory, and heat−clearing properties. Their conservation and sustainable use are of direct relevance to traditional Chinese medicine resources.

(3) Data Availability and Modeling Reliability: Reliable occurrence records were obtained from the Chinese Virtual Herbarium (CVH), the Global Biodiversity Information Facility (GBIF), and published literature. Each species possesses >100 georeferenced occurrence points, satisfying the minimum sample−size requirement (≥30 records) for robust MaxEnt modeling. Many other Gentiana species have too few or imprecise occurrence data to support quantitative distribution modeling. (4) Climate−Response Contrast: Preliminary analyses indicated distinct responses of the three species to key climatic drivers: *G. rhodantha* is highly sensitive to winter minimum temperature, *G. cephalantha* to temperature seasonality, and *G. rigescens* to precipitation seasonality. Studying these contrasting responses provides a framework for understanding the genus−level adaptation strategies under climate change. By focusing on species that are ecologically representative, medicinally important, data−sufficient, and exhibit differential climate sensitivity, this study aims to establish a transferable methodology for climate−risk assessment and conservation planning for the entire *Gentiana* genus.

### 2.2 Species occurrence data

**Figure 1:**
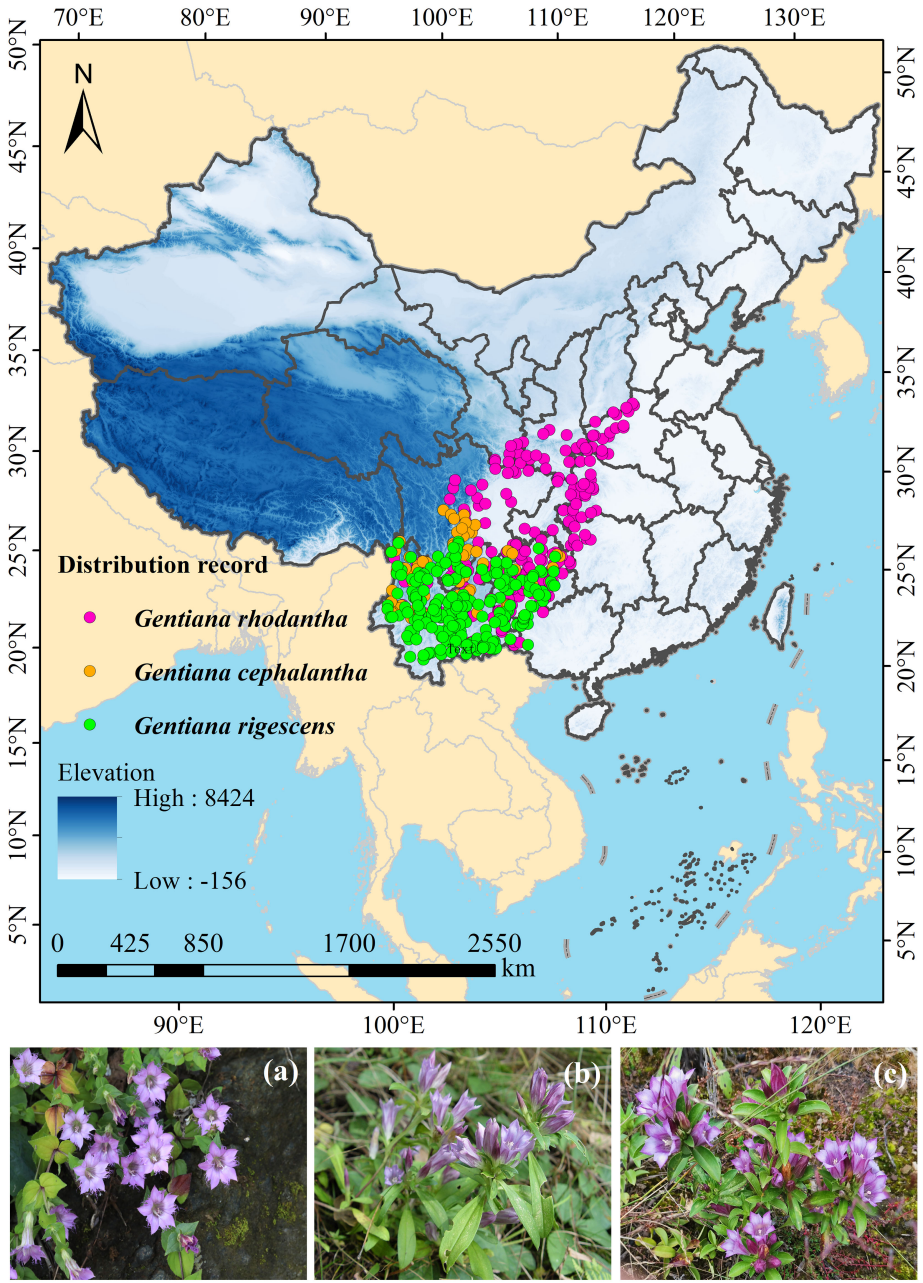
The occurrence records of three *Gentiana* species in China. Plant morphological characteristics of three *Gentiana* species **(a)***Gentiana rhodantha*, **(b)***Gentiana cephalantha*, and **(c)***Gentiana rigescens*.

The latitude and longitude coordinates of three *Gentiana* species distribution sites in China were collected through the following approaches: (1) searching the Chinese Virtual Herbarium (CVH, https://www.cvh.ac.cn) and the Global Biodiversity Information Facility (GBIF, https://www.gbif.org); (2) reviewing and collating relevant literature on the target plants. For occurrence records where only administrative village names were provided as location information, latitude and longitude data were converted by using the platform (https://map.yanue.net/). The map of China used in this study was sourced from the Standard Map Service Website of the Ministry of Natural Resources of China (http://bzdt.ch.mnr.gov.cn), with the map approval number: GS (2023) 2762. Finally, occurrence data for 303 *G. rhodantha*, 101 *G. cephalantha*, and 240 *G. rigescens* individuals were obtained in China (**Figure 1**).

### 2.3 Environmental data

#### 2.3.1 Data source and scenarios

Current climate data, future climate data (covering three periods: 2041–2060, 2061–2080, and 2081–2100) including 19 bioclimatic factors, and topographic data (elevation) were all obtained from the WorldClim (https://www.worldclim.org/). Two shared socioeconomic pathways (SSPs) were selected: SSP126 (sustainable development pathway) and SSP585 (fossil fuel-dominated development pathway). The inclusion of future climate periods [2041–2060 (2050s), 2061–2080 (2070s), and 2081–2100 (2090s)] ensures that the simulation results are more consistent with real-world conditions (Du, 2022). Under the context of global warming intensification, frequent extreme weather events have severely impacted ecosystems and posed significant challenges to socioeconomic systems; simulation results based on these SSP scenarios have been widely applied in predicting extreme climate events, ecosystem changes, and impacts on hydrological cycles. Based on the future climate data under SSP126 and SSP585 scenarios, the 19 climatic factors and topographic data (elevation) were clipped using the geographic information system software ArcGIS 10.8 (**Table 1**), providing a data basis for in-depth simulation and analysis.

**Table 1:** Twenty environmental variables.

Variables	Description	Units
Bio1	Annual mean temperature	°C
Bio2	Mean diurnal range (Mean of monthly)	°C
Bio3	Isothermality (Bio2/Bio7) (× 100)	
Bio4	Standard deviation of temperature seasonality	
Bio5	Max temperature of warmest month	°C
Bio6	Min temperature of coldest month	°C
Bio7	Temperature annual range (Bio5-Bio6)	°C
Bio8	Mean temperature of wettest quarter	°C
Bio9	Mean temperature of driest quarter	°C
Bio10	Mean temperature of warmest quarter	°C
Bio11	Mean temperature of coldest quarter	°C
Bio12	Annual precipitation	mm
Bio13	Precipitation of wettest month	mm
Bio14	Precipitation of driest month	mm
Bio15	Variation of precipitation seasonality	
Bio16	Precipitation of wettest quarter	mm
Bio17	Precipitation of driest quarter	mm
Bio18	Precipitation of warmest quarter	mm
Bio19	Precipitation of coldest quarter	mm
Altitude	Altitude	m

#### 2.3.2 Data processing

Specimens lacking detailed geographical coordinates and duplicate records were excluded, and the three *Gentiana* species were georeferenced by their precise longitude and latitude coordinates. To mitigate overfitting caused by spatially clustered occurrence points, a 5 km × 5 km grid system (equivalent to a spatial resolution of 2.5’) was constructed using ArcGIS 10.8. This resolution was chosen to balance spatial precision with ecological relevance, as it approximates the typical scale of habitat heterogeneity in the mountainous regions where Gentiana species occur, and aligns with the resolution of widely used climate datasets such as WorldClim. When multiple occurrence points fell within a single grid cell, only the point closest to the grid centroid was retained, with redundant points removed through spatial thinning. This process yielded 303 valid sampling points for *Gentiana rhodantha*, 101 for *Gentiana cephalantha*, and 240 for *Gentiana rigescens* for subsequent modeling analyses. MaxEnt 3.4.4 (http://biodiversityinformatics.amnh.org/open_source/maxent/, accessed 18 May 2025) was employed to analyze the distribution-influencing factors of the three *Gentiana* species. The valid occurrence data obtained from ArcGIS 10.8 were imported into IBM SPSS Statistics 26 for correlation analysis, examining 19 bioclimatic variables. Using a correlation coefficient threshold of >0.75, variables with biological significance and high contribution rates in initial model runs were retained to reduce multicollinearity-induced overfitting (**Table 2**).

**Table 2:** Screening of climatic variables influencing the potential geographical distribution of three *Gentiana* species.

Environment variable	Description	Unit	Weather to use *G. rhodantha* for Modeling	Weather to use *G. cephalantha* for Modeling	Weather to use *G. rigescens* for Modeling
Bio1	Annual mean temperature	°C	No	No	No
Bio2	Mean diurnal range	°C	No	No	Yes
Bio3	Isothermality	%	Yes	No	No
Bio4	Temperature seasonality	—	No	Yes	Yes
Bio5	Max. temperature of warmest month	°C	No	No	No
Bio6	Min. temperature of coldest month	°C	Yes	No	No
Bio7	Temperature annual range	°C	Yes	No	No
Bio8	Mean temperature of wettest quarter	°C	No	No	No
Bio9	Mean temperature of driest quarter	°C	No	No	Yes
Bio10	Mean temperature of warmest quarter	°C	No	No	No
Bio11	Mean temperature of coldest quarter	°C	No	Yes	No
Bio12	Annual precipitation	mm	Yes	Yes	Yes
Bio13	Precipitation of wettest month	mm	No	No	No
Bio14	Precipitation of driest month	mm	No	Yes	Yes
Bio15	Precipitation seasonality	—	Yes	Yes	Yes
Bio16	Precipitation of wettest quarter	mm	No	No	No
Bio17	Precipitation of driest quarter	mm	No	No	No
Bio18	Precipitation of warmest quarter	mm	No	No	No
Bio19	Precipitation of coldest quarter	mm	No	No	No
Altitude	—	m	Yes	Yes	Yes

### 2.4 Model calibration

Occurrence data and environmental variables for 303 *G. rhodantha*, 101 *G. cephalantha*, and 240 *G. rigescens* individuals were imported into MaxEnt 3.4.4. The model was configured with a 75% training data and 25% testing data split, a maximum of 10,000 iterations, 10 replicate runs, and cross-validation with random data partitioning. Jackknife tests were conducted to evaluate the contribution of each environmental variable to the distribution models, with output formatted as logistic probability surfaces (.asc files). These outputs were imported into ArcGIS 10.8, and reclassification tools were used to map potential suitable habitats for the three *Gentiana* species under current and future climate scenarios.

### 2.5 Model accuracy assessment

The area under the receiver operating characteristic (ROC) curve (AUC) served as the primary metric for evaluating model performance. Since AUC values provide a threshold-independent measure of model performance, they offer robust evaluation results (Jiménez-Valverde, 2014; Huang et al., 2021). Higher AUC values indicate superior model performance: values between 0.5-0.7 denote poor performance, 0.7-0.9 indicate moderate performance, and values >0.9 signify good model performance. In addition to the AUC evaluation, model predictive performance was further assessed using the True Skill Statistic (TSS). This metric combines sensitivity and specificity to provide a balanced assessment that accounts for potential sample imbalance in species occurrence records (Lu et al., 2025; Wu et al., 2025). TSS scores range from −1 to 1, where values approaching 1 indicate higher predictive accuracy, and scores between 0.6 and 1 are typically classified as representing good model performance. All TSS computations in this study were conducted in R version 4.2.1. Dominant environmental factors were identified based on MaxEnt 3.4.4 output metrics, including variable contribution rates and permutation importance values, combined with regularized training gain results from Jackknife tests.

### 2.6 Environmental factor importance evaluation and dominant factor screening

Relative contribution rates quantify the explanatory weight of each variable, while permutation importance values reflect its stable contribution after random permutation of training data. Higher values for either metric indicate greater importance of the variable in driving the species’ potential distribution. The Jackknife test in MaxEnt was used to evaluate the relative importance of each environmental variable. This procedure iteratively constructs models by omitting one variable at a time and also uses each variable in isolation, thereby assessing its unique contribution to the model’s predictive performance (Li et al., 2016).

### 2.7 Dynamic changes and migration trends of suitable habitats

MaxEnt 3.4.4 was used to generate potential geographical distributions of the three *Gentiana* species under current and future scenarios. Using ArcGIS 10.8 data conversion tools, model outputs were converted to TIF-format raster data. Based on natural breaks classification and consideration of actual species distribution patterns, the study area was categorized into four suitability classes: non-suitable (0-0.1), low-suitable (0.1-0.3), medium-suitable (0.3-0.5), and high-suitable (0.5-1) for visualization and analysis.

ArcGIS 10.8 was used to visualize MaxEnt outputs, with the Reclassify tool applied to quantify areas within each suitability class. A suitability threshold of 0.10 was determined by using the sensitivity-specificity sum maximization approach, defining areas with species occurrence probabilities <0.10 as non-suitable. In ArcGIS 10.8, areas with suitability ≥0.1 (encompassing low, medium, and high suitability classes) were designated as suitable habitats, while those with <0.1 were classified as non-suitable. These data were visualized to map the spatial patterns of suitable habitats, showing retained, lost, and newly gained areas, with statistical analyses of habitat expansion, contraction, and stability.

## 3 Results

### 3.1 Evaluation of MaxEnt model reliability

Based on the correlation analysis results between environmental factors and occurrence data, longitude/latitude data for the three Gentiana species and selected environmental variables were input into MaxEnt 3.4.4 for ten iterative simulations. The resulting AUC values were 0.944 for *G. rhodantha*, 0.964 for *G. cephalantha*, and 0.968 for *G. rigescens*. AUC values for all training datasets exceeded 0.9, indicating high predictive accuracy for the constructed models. In addition, the model performance was further evaluated using the True Skill Statistic (TSS), which provides a balanced assessment by combining sensitivity and specificity. The calculated TSS values were 0.839 ± 0.007 for *G. rhodantha*, 0.862 ± 0.009 for *G. cephalantha*, and 0.904 ± 0.005 for *G. rigescens*. All TSS scores fell within the range of 0.6–1.0, which is classified as representing good to excellent model performance. The combined high AUC and TSS values ensure reliable ecological suitability assessments for these Gentiana species.

### 3.2 Dominant environmental variables affecting *Gentiana* distributions

**Figure 2:**
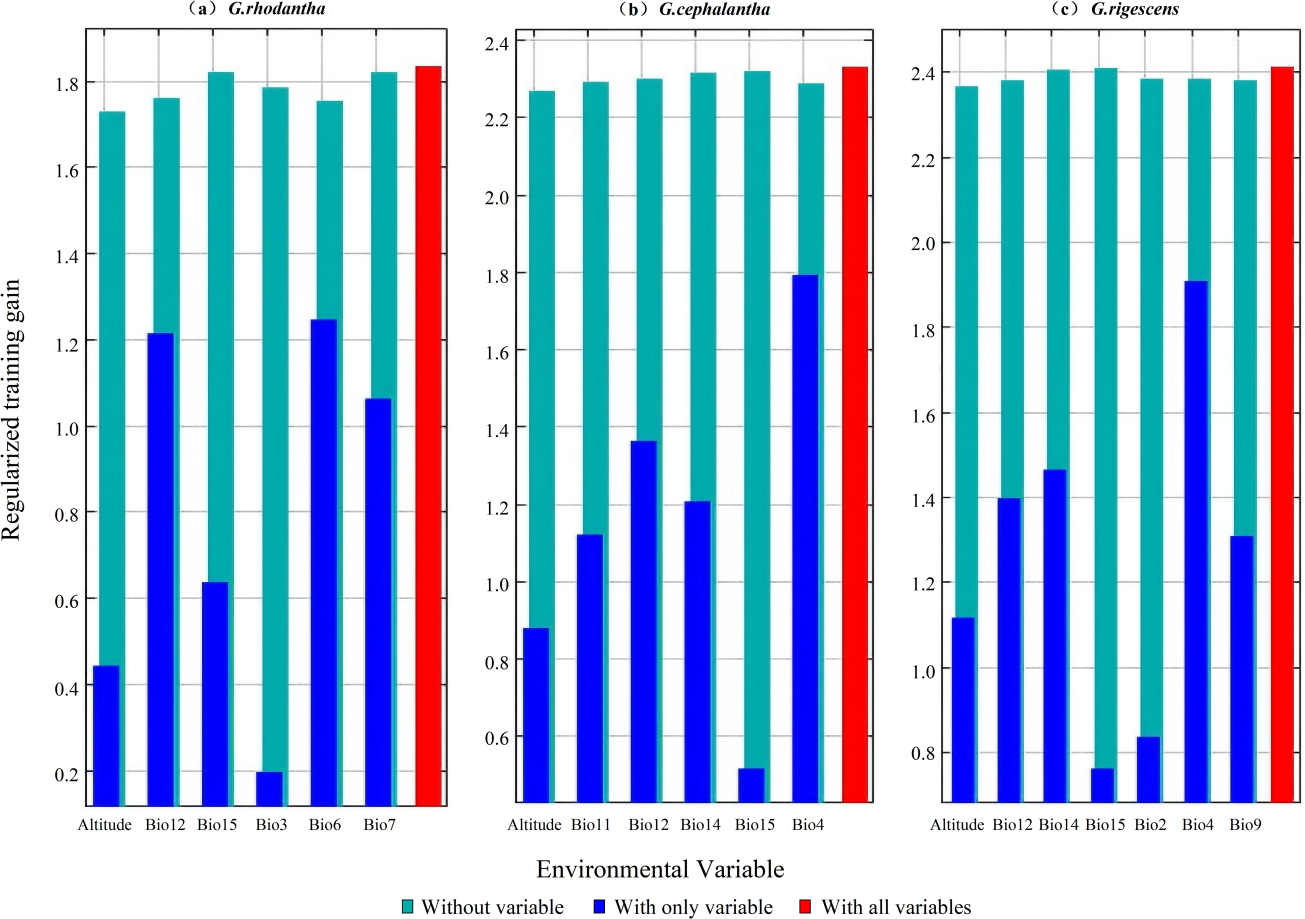
Jackknife test scores evaluating the importance of screened environmental factors for the potential distribution prediction models of three *Gentiana* species. **(a)***G.rhodantha*; **(b)***G.cephalantha*; **(c)***G.rigescens*. The colored bars represent three Jackknife test scenarios: teal bars = model performance when the corresponding environmental variable is excluded (Without variable); blue bars = model performance when only the corresponding environmental variable is included (With only variable); red bars = model performance when all environmental variables are included (With all variables). Environmental variables on the x-axis include altitude (Altitude) and bioclimatic factors (e.g., annual precipitation, precipitation seasonality, and temperature-related indices).

**Figure 3:**
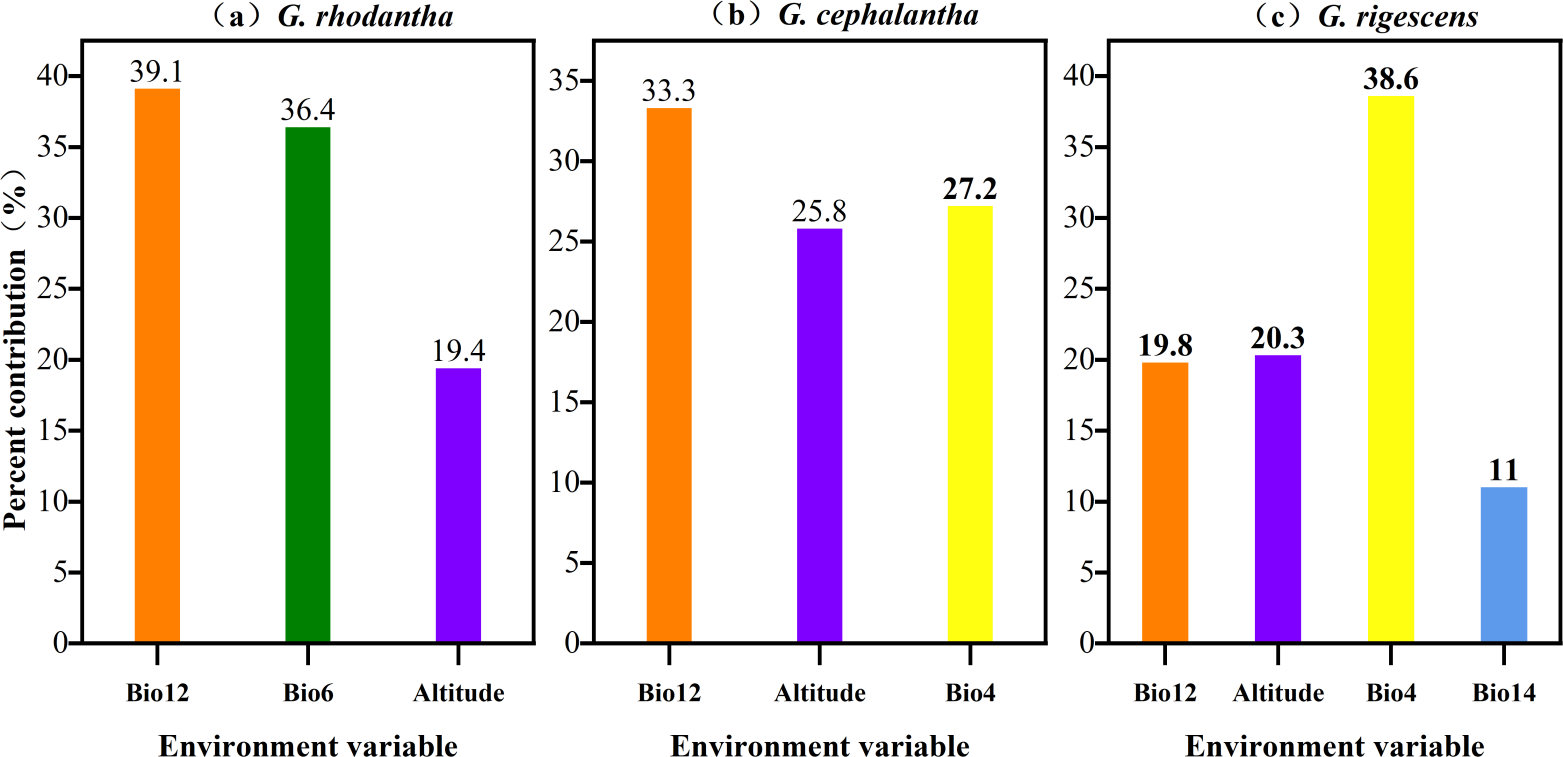
Contribution degrees of dominant environmental variables for three species of *Gentiana***(a)***G.rhodantha*; **(b)***G.cephalantha*; **(c)***G.rigescens*. The colored bars represent the percentage contribution of each key environmental variable to the species' potential distribution model: **(a)** orange bar = Bio12 (annual precipitation, 39.1%), green bar = Bio6 (minimum temperature of coldest month, 36.4%), purple bar = Altitude (19.4%); **(b)** orange bar = Bio12 (annual precipitation, 33.3%), purple bar = Altitude (25.8%), yellow bar = Bio4 (temperature seasonality, 27.2%); **(c)** orange bar = Bio12 (annual precipitation, 19.8%), purple bar = Altitude (20.3%), yellow bar = Bio4 (temperature seasonality, 38.6%), blue bar = Bio14 (precipitation of driest month, 11.0%).

Dominant environmental variables influencing suitable habitats were identified from MaxEnt-derived contribution rates, permutation importance values, and Jackknife test regularized training gains (**Figures 2**, **3**). Key climatic factors affecting current potential distribution of *G. rhodantha* include: mean annual precipitation (Bio12, 39.1%), minimum temperature of the coldest month (Bio6, 36.4%), altitude (19.4%), isothermality (Bio3, 2.4%), precipitation seasonality (Bio15, 2%), and annual temperature range (Bio7, 0.7%). Cumulatively, mean annual precipitation, minimum temperature of the coldest month, and altitude accounted for 94.9% of model variance, representing the primary drivers of *G. rhodantha* distribution.

For *G. cephalantha*, major influencing factors include: mean annual precipitation (Bio12, 33.3%), temperature seasonality (Bio4, 27.2%), altitude (25.8%), mean temperature of the coldest quarter (Bio11, 8.8%), precipitation of the driest month (Bio14, 4.1%), and precipitation seasonality (Bio15, 0.8%). Mean annual precipitation, temperature seasonality, and altitude collectively contributed 86.3% to the model, forming the dominant factors shaping *G. cephalantha* distribution. Primary factors affecting *G. rigescens* distribution include: temperature seasonality (Bio4, 38.6%), altitude (20.3%), mean annual precipitation (Bio12, 19.8%), precipitation of the driest month (Bio14, 11%), mean temperature of the driest quarter (Bio9, 8.5%), mean diurnal range (Bio2, 1.2%), and precipitation seasonality (Bio15, 0.5%). Temperature seasonality, altitude, mean annual precipitation, and precipitation of the driest month together accounted for 89.7% of model variance, representing the key determinants of *G. rigescens* distribution.

### 3.3 Distribution of *Gentiana* species under current climate conditions

**Figure 4:**
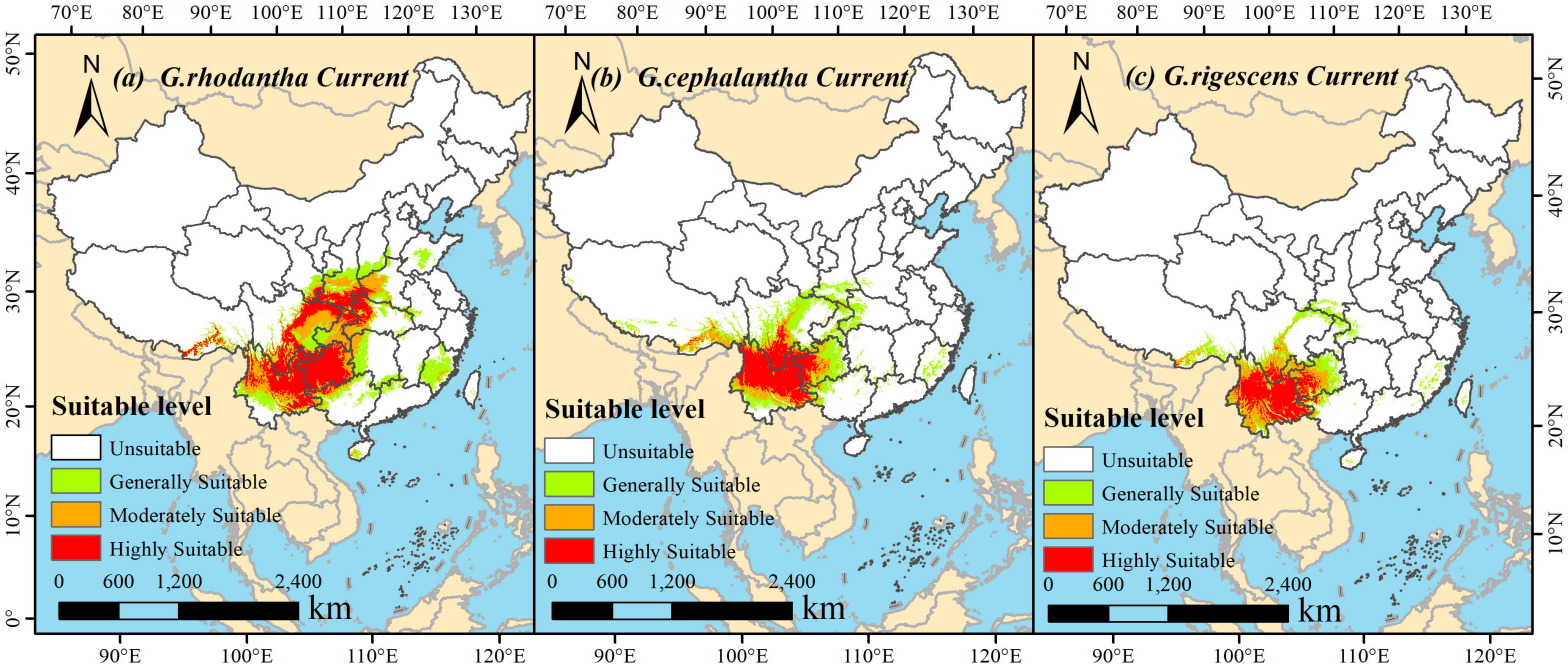
Schematic map of suitable areas of three *Gentiana* species in China under current climatic conditions. **(a)***G.rhodantha*; **(b)***G.cephalantha*; **(c)***G.rigescens*. The color gradient represents four levels of habitat suitability: white = Unsuitable, light green = Generally Suitable, orange = Moderately Suitable, red = Highly Suitable. The scale bar at the bottom of each subfigure indicates distances in kilometers, with a north arrow for spatial orientation.

Using ArcGIS 10.8, the results predicted by MaxEnt 3.4.4 were visualized. Under the current climate context, the geographical distribution patterns of the three *Gentiana* species are shown in **Figure 4**, and the area proportions of each suitability class (high, medium, and low) were calculated.

The total suitable area of *Gentiana rhodantha* (red-flowered gentian) reached 145.13 × 10^4^ km^2^, accounting for approximately 15.12% of China’s total land area. For *Gentiana cephalantha* (head-flowered gentian), the total suitable area was 94.92 × 10^4^ km^2^, making up about 9.89% of China’s total land area. Meanwhile, the total suitable area of *Gentiana rigescens* (Yunnan gentian) was 71.41 × 10^4^ km^2^, accounting for roughly 7.44% of China’s total land area. Within China, the highly suitable area of *G. rhodantha* was 52.23 × 10^4^ km^2^, accounting for 5.44% of China’s land area, and it was mainly distributed in Guizhou, Yunnan, Sichuan, Chongqing, Hubei, Shaanxi, western Guangxi, southern Gansu, western Henan, and southern Tibet, among other regions. The medium and low suitable areas of *G. rhodantha* were 40.90 × 10^4^ km^2^ and 51.99 × 10^4^ km^2^, respectively, accounting for 4.26% and 5.42% of China’s land area. For *G. cephalantha*, the highly suitable area was 29.42 × 10^4^ km^2^, accounting for 3.06% of China’s land area, with primary distribution in Yunnan, Sichuan, Guizhou, western Guangxi, and southern Tibet. Its medium and low suitable areas were 19.07 × 10^4^ km^2^ and 46.43 × 10^4^ km^2^, representing 1.99% and 4.84% of China’s land area, respectively. Regarding *G. rigescens*, the highly suitable area was 23.02 × 10^4^ km^2^ (2.40% of China’s land area), mainly distributed in Yunnan, Sichuan, Guizhou, western Guangxi, and southern Tibet. Its medium and low suitable areas were 21.80 × 10^4^ km^2^ and 26.59 × 10^4^ km^2^, accounting for 2.27% and 2.77% of China’s land area, respectively.

### 3.4 Distribution of *Gentiana* species under future climate change

**Figure 5:**
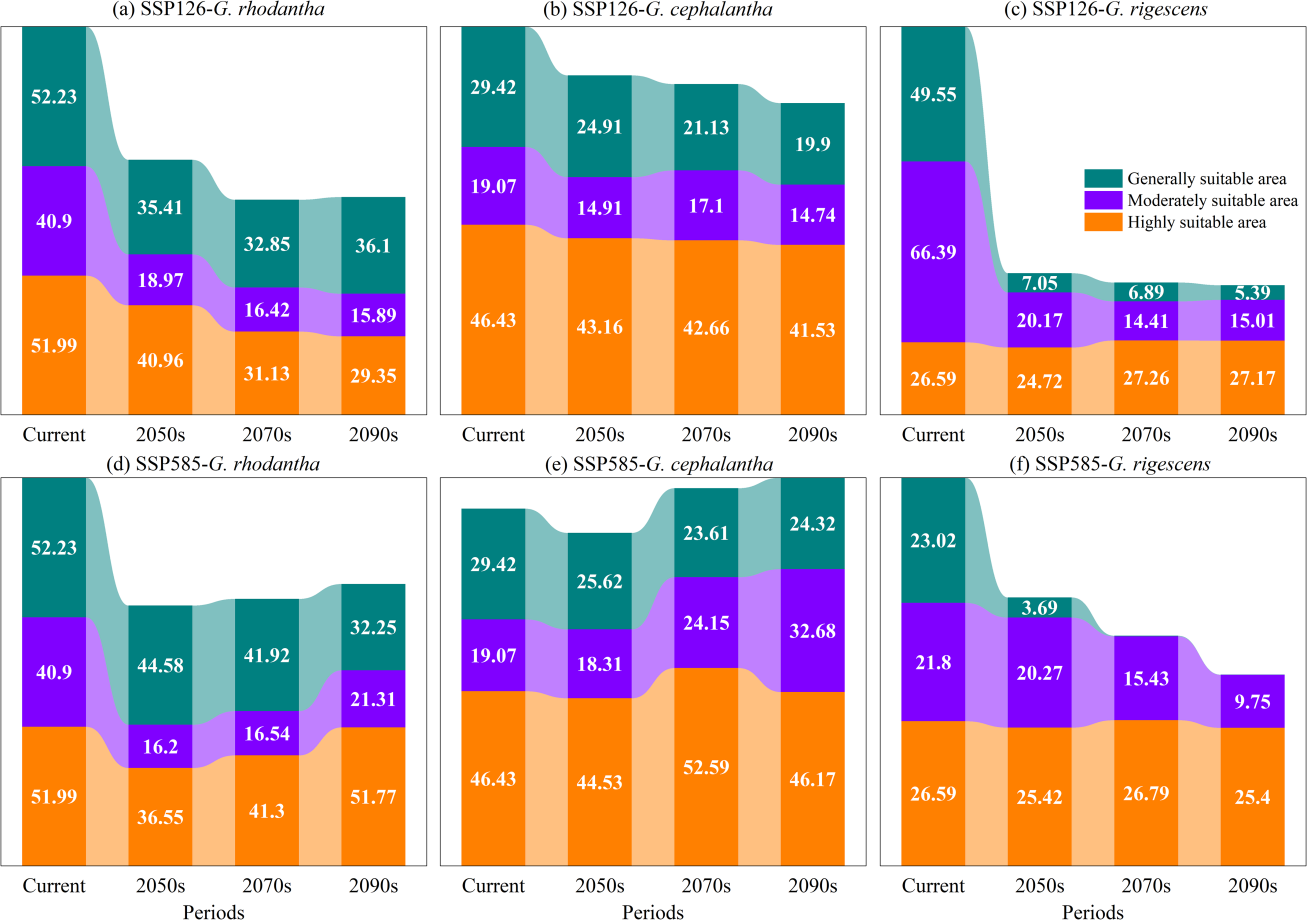
Projected shifts in suitable areas for three *Gentiana* species during different periods under two climate change scenarios (unit: 10^4^ km^2^). **(a)***G. rhodantha*; **(b)***G.cephalantha*; **(c)***G. rigescens*. The top row represents the SSP126 scenario (low emission), and the bottom row represents the SSP585 scenario (high emission). The colored layers represent three suitability levels: teal = Generally suitable area, purple = Moderately suitable area, orange = Highly suitable area. Values in each layer indicate the area (×10^4^km^2^) of that suitability class in the corresponding period (Current, 2050s, 2070s, 2090s).

**Figure 6:**
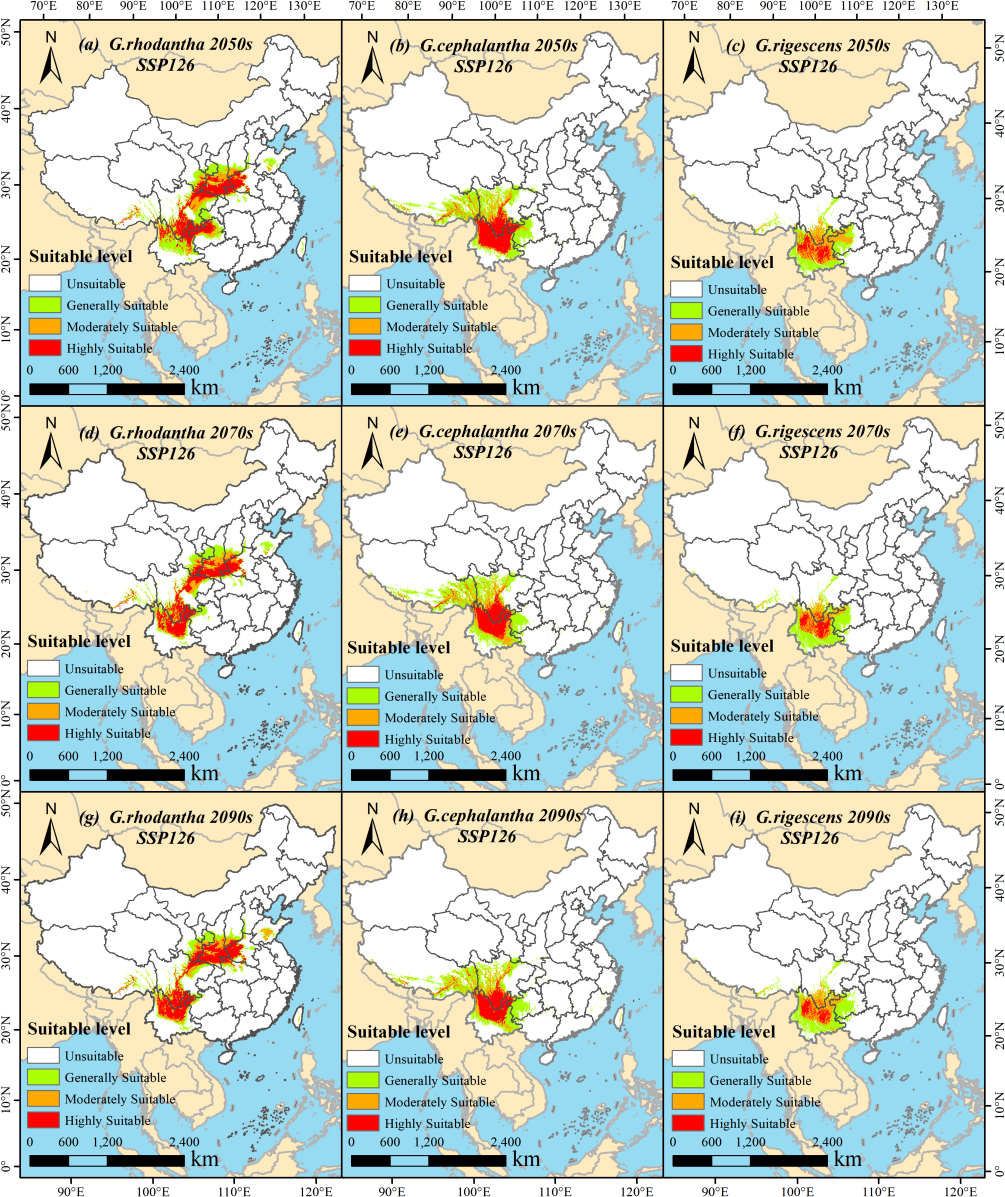
Suitable areas of three *Gentiana* species in China in different periods under the SSP126 (low emission) climate change scenario. **(a, d, g)***G.rhodantha* in the 2050s, 2070s, and 2090s, respectively; **(b, e, h)***G.cephalantha* in the 2050s, 2070s, and 2090s, respectively; **(c, f, i)***G.rigescens* in the 2050s, 2070s, and 2090s, respectively. The color gradient represents four levels of habitat suitability: white = Unsuitable, light green = Generally Suitable, orange = Moderately Suitable, red = Highly Suitable. Each subfigure includes a north arrow for spatial orientation and a scale bar indicating distances in kilometers.

Under the SSP126 climate scenario (a sustainable development pathway emphasizing low-carbon transition and green technological innovation), the total suitable area of *G. rhodantha* (red-flowered gentian) showed an overall decreasing trend. Specifically, its suitable area was 95.34 × 10^4^ km^2^, 80.40 × 10^4^ km^2^, and 81.35 × 10^4^ km^2^ in the periods 2041–2060, 2061–2080, and 2081–2100, respectively. For *G. cephalantha* (head-flowered gentian), the total suitable area also exhibited a decreasing trend, declining to 82.98 × 10^4^ km^2^, 80.88 × 10^4^ km^2^, and 76.18 × 10^4^ km^2^ in 2041–2060, 2061–2080, and 2081–2100, respectively. Meanwhile, the total suitable area of *G. rigescens* (Yunnan gentian) gradually shrank to 51.94 × 10^4^ km^2^, 48.56 × 10^4^ km^2^, and 47.56 × 10^4^ km^2^ in the same three periods. In contrast, under the SSP585 climate scenario (a rapid development pathway with high fossil fuel consumption), the total suitable area of *G. rhodantha* in future periods was reduced compared to the current area; however, it increased to 97.33 × 10^4^ km^2^, 99.76 × 10^4^ km^2^, and 105.34 × 10^4^ km^2^ in 2041–2060, 2061–2080, and 2081–2100, respectively. The total suitable area of *G. cephalantha* mainly showed an upward trend, reaching 88.45 × 10^4^ km^2^, 100.35 × 10^4^ km^2^, and 103.16 × 10^4^ km^2^ in the three periods mentioned above. For *G. rigescens*, the total suitable area continued to decrease, dropping to 49.38 × 10^4^ km^2^, 42.33 × 10^4^ km^2^, and 35.18 × 10^4^ km^2^ in 2041–2060, 2061–2080, and 2081–2100, respectively (**Figures 5**, **6**).

**Figure 7:**
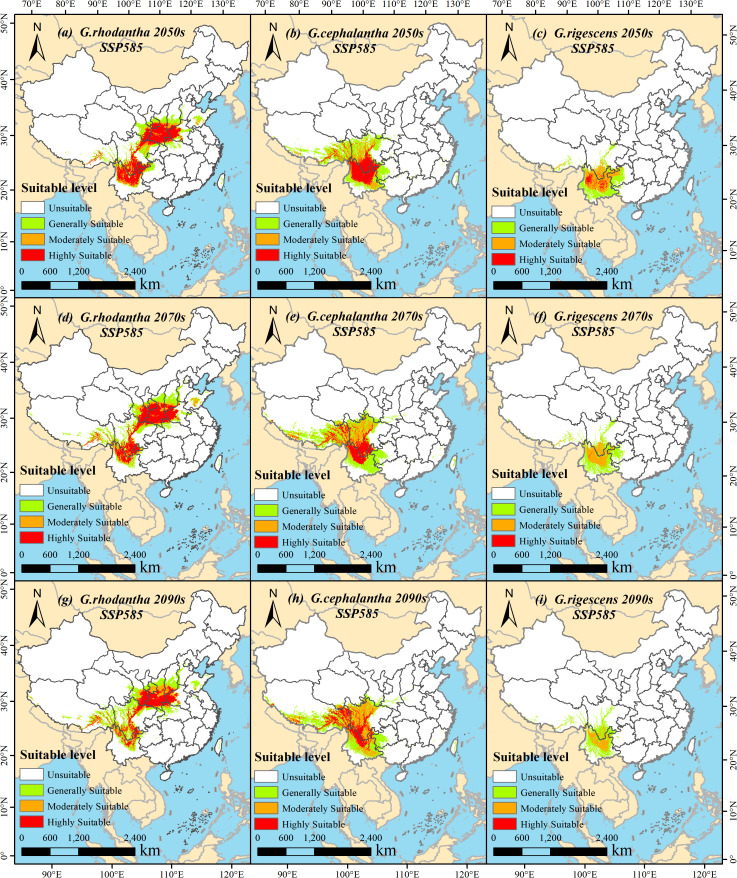
Schematic map of suitable areas of three *Gentiana* species in China in different periods under the SSP585 (high emission) climate change scenario. **(a, d, g)***G.rhodantha* in the 2050s, 2070s, and 2090s, respectively; **(b, e, h)***G.cephalantha* in the 2050s, 2070s, and 2090s, respectively; **(c, f, i)***G.rigescens* in the 2050s, 2070s, and 2090s, respectively. The color gradient represents four levels of habitat suitability: white = Unsuitable, light green = Generally Suitable, orange = Moderately Suitable, red = Highly Suitable. Each subfigure includes a north arrow for spatial orientation and a scale bar indicating distances in kilometers.

Using ArcGIS 10.8 software, the geographical distribution patterns of the three *Gentiana* species predicted by MaxEnt 3.4.4 for different future periods were visualized. Under the SSP126 scenario: the high-suitable area of *G. rhodantha* decreased but showed a slight increase in 2081–2100 compared to 2061–2080; the low-suitable area of *G. rigescens* gradually increased, while its medium-suitable area slightly increased in 2081–2100 relative to 2061–2080, and the high-suitable area continued to decrease. Under the SSP585 scenario: the high-suitable area of *G. rhodantha* significantly decreased, while its low-suitable area showed a slight expansion northward; the high-suitable area of *G. cephalantha* in Yunnan and Guizhou Provinces significantly reduced, with a slight northward expansion of its medium-suitable area; the high-suitable area of *G. rigescens* almost disappeared, and its medium-suitable area gradually decreased (**Figures 5**, **7**).

Notably, under the high-emission SSP585 scenario, the three *Gentiana* species will face severe survival challenges. The deterioration of climatic conditions in the original high-suitable areas has led to the shrinkage of their growth ranges, reflecting the direct impact of climate change on species distribution patterns. This crisis is mainly driven by the dual pressures of increased habitat instability and intensive human activities. Therefore, systematic conservation strategies are urgently needed to mitigate the loss trend of these plant resources.

### 3.5 Characteristics of changes in suitable habitat area under future climate change

**Figure 8:**
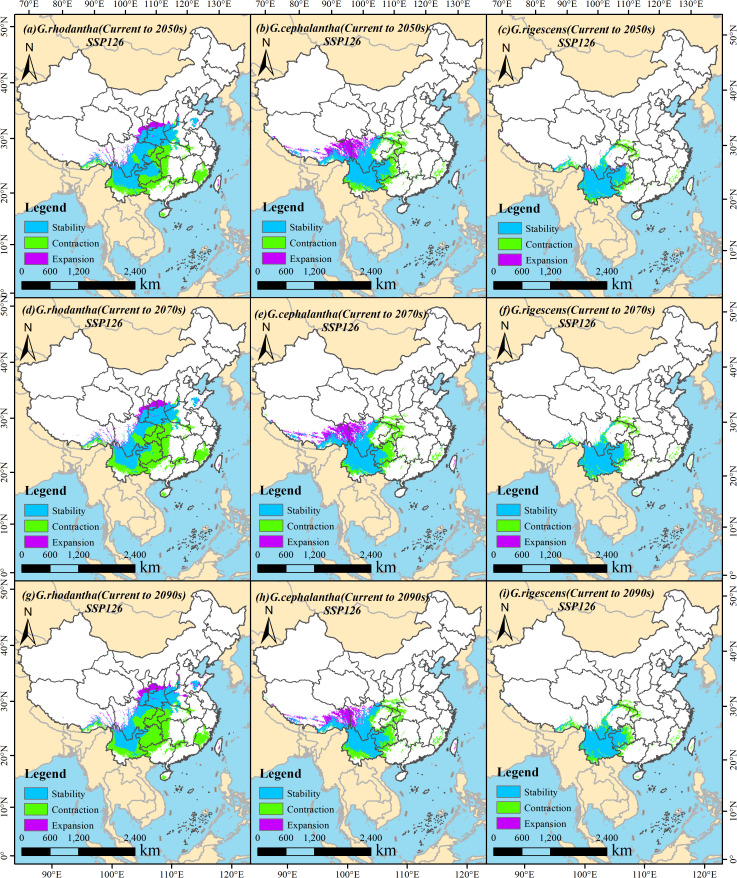
Changes in the suitable habitat area of three *Gentiana* species under the SSP126 (low emission) climate change scenario in the future. **(a, d, g)***G. rhodantha* from Current to 2050s, 2070s, and 2090s, respectively; **(b, e, h)***G.cephalantha* from Current to 2050s, 2070s, and 2090s, respectively; **(c, f, i)***G. rigescens* from Current to 2050s, 2070s, and 2090s, respectively. The colored regions represent four types of habitat change: light blue = Stability (persistently suitable), green = Contraction (loss of suitability), purple = Expansion (gain of suitability). Each subfigure includes a north arrow for spatial orientation and a scale bar indicating distances in kilometers.

**Figure 9:**
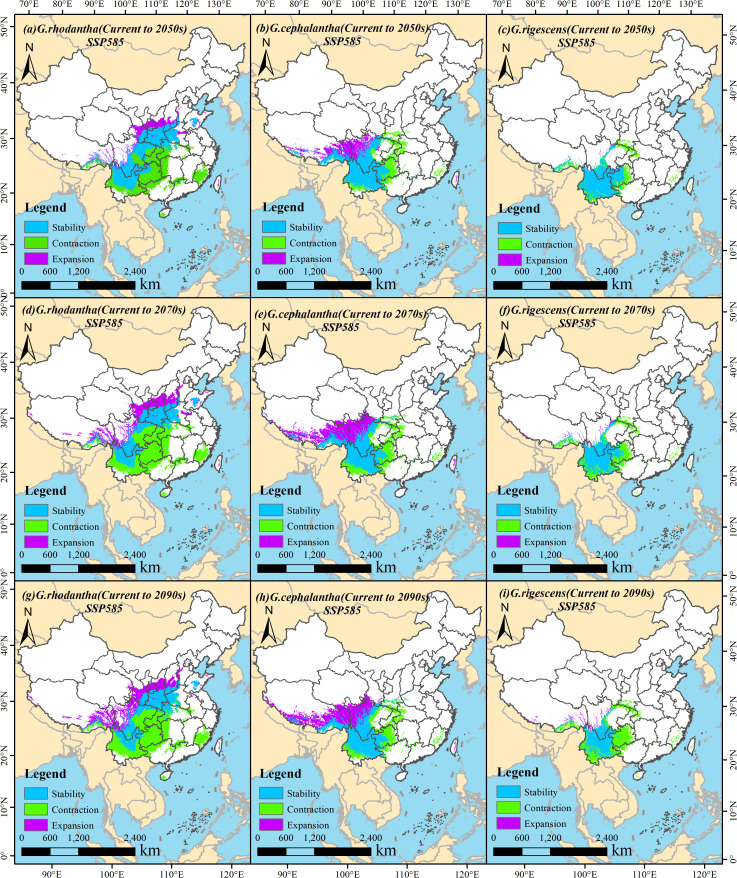
Changes in the suitable habitat area of three *Gentiana* species under the SSP585 (high emission) climate change scenario in the future. **(a, d, g)***G.rhodantha* from Current to 2050s, 2070s, and 2090s, respectively; **(b, e, h)***G.cephalantha* from Current to 2050s, 2070s, and 2090s, respectively; **(c, f, i)***G.rigescens* from Current to 2050s, 2070s, and 2090s, respectively. The colored regions represent three types of habitat change: light blue = Stability (persistently suitable habitat), green = Contraction (habitat that loses suitability), purple = Expansion (newly gained suitable habitat). Each subfigure includes a north arrow for spatial orientation and a scale bar indicating distances in kilometers.

Based on the current distribution of the studied species, this section comparatively analyzes the spatial pattern changes of suitable habitats across different periods under the specified climate scenarios. During 2041–2060 (the 2050s), under the SSP126 scenario, the retained area of suitable habitat for *G. rhodantha* (Honghua Longdan) was 83.01 × 10^4^ km^2^, with a retention rate of 52.81%; the increased area of its suitable habitat was 12.24 × 10^4^ km^2^, with a gain rate of 7.78%; and the lost area of its suitable habitat was 61.93 × 10^4^ km^2^, with a loss rate of 39.40%. For *G. cephalantha* (Touhua Longdan), the retained area of suitable habitat was 62.07 × 10^4^ km^2^, with a retention rate of 53.73%; the increased area was 20.90 × 10^4^ km^2^, with a gain rate of 18.09%; and the lost area was 32.56 × 10^4^ km^2^, with a loss rate of 28.18%. For *G. rigescens* (Dian Longdan), the retained area of suitable habitat was 50.26 × 10^4^ km^2^, with a retention rate of 69.02%; the increased area was 1.63 × 10^4^ km^2^, with a gain rate of 2.24%; and the lost area was 20.93 × 10^4^ km^2^, with a loss rate of 28.74% (**Figure 8**). Under the SSP585 scenario, the retained area of suitable habitat for *G. rhodantha* was 74.81 × 10^4^ km^2^, with a retention rate of 44.71%; the increased area was 22.40 × 10^4^ km^2^, with a gain rate of 13.38%; and the lost area was 70.13 × 10^4^ km^2^, with a loss rate of 41.91%. For *G. cephalantha*, the retained area of suitable habitat was 63.07 × 10^4^ km^2^, with a retention rate of 52.57%; the increased area was 25.33 × 10^4^ km^2^, with a gain rate of 21.12%; and the lost area was 31.56 × 10^4^ km^2^, with a loss rate of 26.31%. For *G. rigescens*, the retained area of suitable habitat was 48.25 × 10^4^ km^2^, with a retention rate of 66.66%; the increased area was 1.18 × 10^4^ km^2^, with a gain rate of 1.63%; and the lost area was 22.95 × 10^4^ km^2^, with a loss rate of 31.71% (**Figure 9**; **Table 3**).

**Table 3:** Suitable habitat area changes of three *Gentiana* species under future climate change.

Species	Period	Area (10^4^ km^2^)			Rate of change (%)		
		Stability	Expansion	Contraction	Stability	Expansion	Contraction
*G. rhodantha*	SSP126(2041-2060)	83.01	12.24	61.93	52.81	7.78	39.40
	SSP126(2061-2080)	67.98	12.35	76.96	43.22	7.85	48.93
	SSP126(2081-2100)	66.46	14.72	78.47	41.63	9.22	49.15
	SSP585(2041-2060)	74.81	22.40	70.13	44.71	13.38	41.91
	SSP585(2061-2080)	61.42	38.36	83.52	33.50	20.93	45.57
	SSP585(2081-2100)	60.41	45.02	84.54	31.80	23.70	44.50
*G. cephalantha*	SSP126(2041-2060)	62.07	20.90	32.56	53.73	18.09	28.18
	SSP126(2061-2080)	59.12	21.78	35.52	50.78	18.71	30.51
	SSP126(2081-2100)	58.45	17.65	36.19	52.06	15.72	32.23
	SSP585(2041-2060)	63.07	25.33	31.56	52.57	21.12	26.31
	SSP585(2061-2080)	56.66	43.48	37.97	41.03	31.48	27.49
	SSP585(2081-2100)	57.89	45.06	36.74	41.44	32.26	26.30
*G. rigescens*	SSP126(2041-2060)	50.26	1.63	20.93	69.02	2.24	28.74
	SSP126(2061-2080)	47.81	0.77	23.36	66.45	1.07	32.47
	SSP126(2081-2100)	46.97	0.57	24.19	65.48	0.80	33.73
	SSP585(2041-2060)	48.25	1.18	22.95	66.66	1.63	31.71
	SSP585(2061-2080)	39.55	2.61	31.63	53.59	3.54	42.87
	SSP585(2081-2100)	30.65	4.33	40.51	40.61	5.73	53.66

During 2061–2080 (the 2070s), under the SSP126 scenario, the retained area of suitable habitat for *G. rhodantha* was 67.98 × 10^4^ km^2^, with a retention rate of 43.22%; the increased area was 12.35 × 10^4^ km^2^, with a gain rate of 7.85%; and the lost area was 76.96 × 10^4^ km^2^, with a loss rate of 48.93%. For *G. cephalantha*, the retained area of suitable habitat was 59.12 × 10^4^ km^2^, with a retention rate of 50.78%; the increased area was 21.78 × 10^4^ km^2^, with a gain rate of 18.71%; and the lost area was 35.52 × 10^4^ km^2^, with a loss rate of 30.51%. For *G. rigescens*, the retained area of suitable habitat was 47.81 × 10^4^ km^2^, with a retention rate of 66.45%; the increased area was 0.77 × 10^4^ km^2^, with a gain rate of 1.07%; and the lost area was 23.36 × 10^4^ km^2^, with a loss rate of 32.47% (**Figure 8**). Under the SSP585 scenario, the retained area of suitable habitat for *G. rhodantha* was 61.42 × 10^4^ km^2^, with a retention rate of 33.50%; the increased area was 38.36 × 10^4^ km^2^, with a gain rate of 20.93%; and the lost area was 83.52 × 10^4^ km^2^, with a loss rate of 45.57%. For *G. cephalantha*, the retained area of suitable habitat was 56.66 × 10^4^ km^2^, with a retention rate of 41.03%; the increased area was 43.48 × 10^4^ km^2^, with a gain rate of 31.48%; and the lost area was 37.97 × 10^4^ km^2^, with a loss rate of 27.49%. For *G. rigescens*, the retained area of suitable habitat was 39.55 × 10^4^ km^2^, with a retention rate of 53.59%; the increased area was 2.61 × 10^4^ km^2^, with a gain rate of 3.54%; and the lost area was 31.63 × 10^4^ km^2^, with a loss rate of 42.87% (**Figure 9**; **Table 3**).

During 2081–2100 (the 2090s), under the SSP126 scenario, the retained area of suitable habitat for *G. rhodantha* was 66.46 × 10^4^ km^2^, with a retention rate of 41.63%; the increased area was 14.72 × 10^4^ km^2^, with a gain rate of 9.22%; and the lost area was 78.47 × 10^4^ km^2^, with a loss rate of 49.15%. For *G. cephalantha*, the retained area of suitable habitat was 58.45 × 10^4^ km^2^, with a retention rate of 52.06%; the increased area was 17.65 × 10^4^ km^2^, with a gain rate of 15.72%; and the lost area was 36.19 × 10^4^ km^2^, with a loss rate of 32.23%. For *G. rigescens*, the retained area of suitable habitat was 46.97 × 10^4^ km^2^, with a retention rate of 65.48%; the increased area was 0.57 × 10^4^ km^2^, with a gain rate of 0.80%; and the lost area was 24.19 × 10^4^ km^2^, with a loss rate of 33.73%. Under the SSP585 scenario, the retained area of suitable habitat for *G. rhodantha* was 60.41 × 10^4^ km^2^, with a retention rate of 31.80%; the increased area was 45.02 × 10^4^ km^2^, with a gain rate of 23.70%; and the lost area was 84.54 × 10^4^ km^2^, with a loss rate of 44.50%. For *G. cephalantha*, the retained area of suitable habitat was 57.89 × 10^4^ km^2^, with a retention rate of 41.44%; the increased area was 45.06 × 10^4^ km^2^, with a gain rate of 32.26%; and the lost area was 36.74 × 10^4^ km^2^, with a loss rate of 26.30%. For *G. rigescens*, the retained area of suitable habitat was 30.65 × 10^4^ km^2^, with a retention rate of 40.61%; the increased area was 4.33 × 10^4^ km^2^, with a gain rate of 5.73%; and the lost area was 40.51 × 10^4^ km^2^, with a loss rate of 53.66% (**Figure 9**; **Table 3**).

### 3.6 Migration of the centroid of *Gentiana* plants under different climatic scenarios

**Figure 10:**
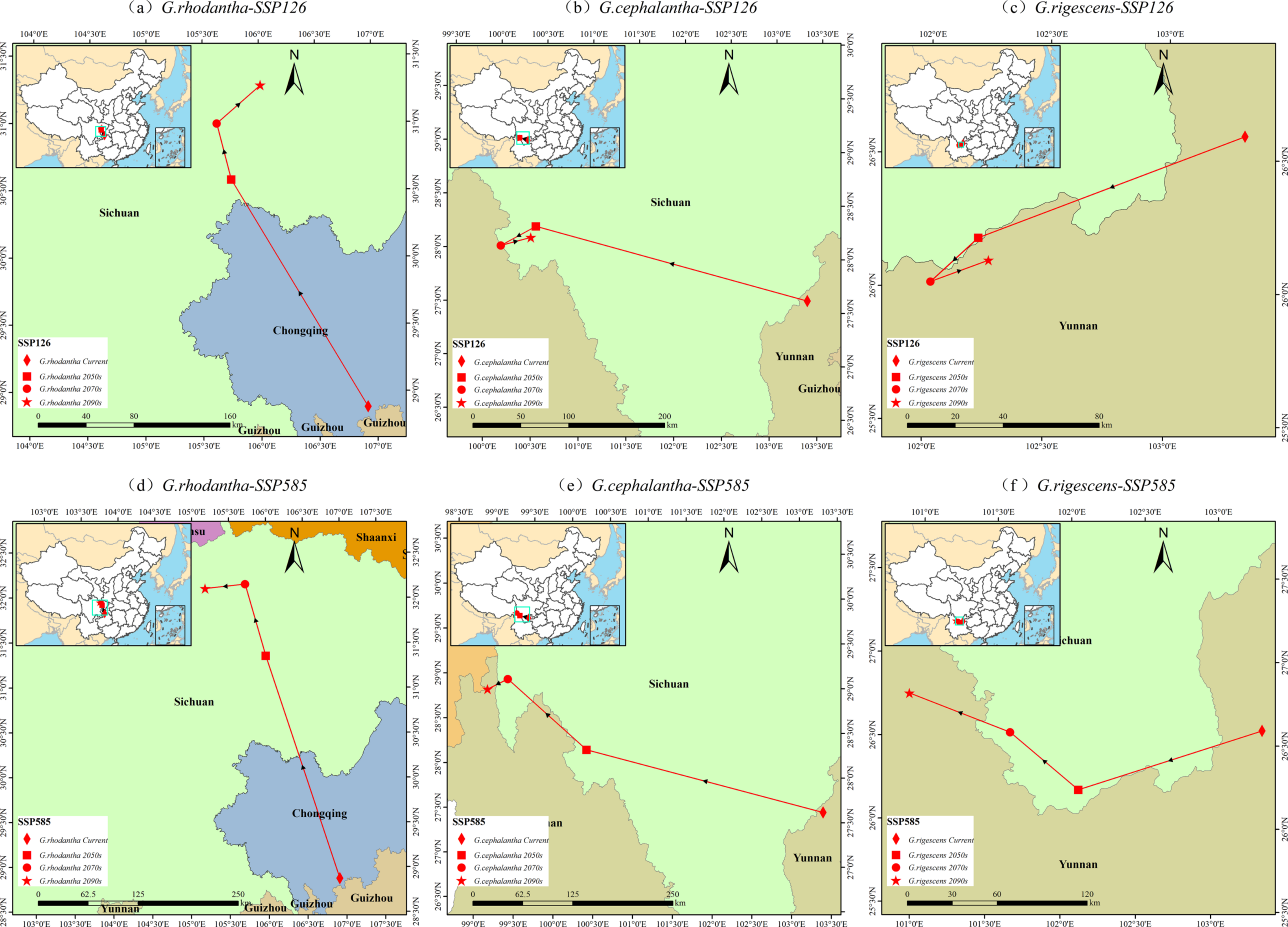
Centroid migration map of three *Gentiana* species under two climate change scenarios. **(a, d)***G. rhodantha* under the SSP126 (low emission) and SSP585 (high emission) scenarios, respectively; **(b, e)***G. cephalantha* under the SSP126 (low emission) and SSP585 (high emission) scenarios, respectively; **(c, f)***G. rigescens* under the SSP126 (low emission) and SSP585 (high emission) scenarios, respectively Different red symbols represent the species' distribution centroid at distinct time points: red circles = Current, red diamonds = 2050s, red triangles = 2070s, red five-pointed stars = 2090s. Red lines connect the symbols to indicate the migration trajectory of the centroid. Each subfigure includes a north arrow for spatial orientation, a scale bar indicating distances in kilometers, and an inset map showing the overall location of the study area in China.

Using ArcGIS 10.8 software, the changes in the distribution centroid from 2041 to 2100 were calculated, and the overall trend of range changes was detected, resulting in the centroid migration path map of *Gentiana* plants (**Figure 10**). Under the SSP126 climate scenario, the current distribution center of *G. rhodantha* is in Chongqing Municipality (106°55′E, 28°53′N); from 2041 to 2060, it is in Suining City, Sichuan Province (105°45′E, 30°35′N); from 2061 to 2080, it is in Nanchong City, Sichuan Province (105°38′E, 31°0′N); and from 2081 to 2100, it remains in Nanchong City, Sichuan Province (106°1′E, 31°17′N); the current distribution center of *G. cephalantha* is in Zhaotong City, Yunnan Province (103°23′E, 27°37′N); from 2041 to 2060, it is in Liangshan Yi Autonomous Prefecture, Sichuan Province (100°28′E, 28°14′N); from 2061 to 2080, it is in Liangshan Yi Autonomous Prefecture, Sichuan Province (100°6′E, 28°2′N); and from 2081 to 2100, it stays in Liangshan Yi Autonomous Prefecture, Sichuan Province (100°25′E, 28°8′N); in general, the distribution centers of *G. rhodantha* and *G. cephalantha* move westward and northward; the current distribution center of *Gentiana* rigescens is in Qujing City, Yunnan Province (103°19′E, 26°35′N); from 2041 to 2060, it is in Liangshan Yi Autonomous Prefecture, Sichuan Province (102°13′E, 26°11′N); from 2061 to 2080, it is in Chuxiong Yi Autonomous Prefecture, Yunnan Province (102°1′E, 26°1′N); and from 2081 to 2100, it remains in Chuxiong Yi Autonomous Prefecture, Yunnan Province (102°16′E, 26°6′N), with its distribution center moving southward and westward, while during the 2070s–2090s, the distribution centers of *G. cephalantha* and *G. rigescens* move eastward. Under the SSP585 climate scenario, the current distribution center of *G. rhodantha* is in Chongqing Municipality (106°55′E, 28°53′N); from 2041 to 2060, it is in Nanchong City, Sichuan Province (105°59′E, 31°23′N); from 2061 to 2080, it is in Guangyuan City, Sichuan Province (105°43′E, 32°11′N); and from 2081 to 2100, it is in Mianyang City, Sichuan Province (105°11′E, 32°8′N); the current distribution center of *G. cephalantha* is in Zhaotong City, Yunnan Province (103°23′E, 27°37′N); from 2041 to 2060, it is in Daocheng County, Ganzi Tibetan Autonomous Prefecture, Sichuan Province (100°19′E, 28°14′N); from 2061 to 2080, it is in Derong County, Ganzi Tibetan Autonomous Prefecture, Sichuan Province (99°16′E, 28°58′N); and from 2081 to 2100, it is in Diqing Tibetan Autonomous Prefecture, Yunnan Province (99°1′E, 28°51′N); in general, the distribution centers of *G. rhodantha* and *G. cephalantha* move westward and northward; the current distribution center of *G. rigescens* is in Qujing City, Yunnan Province (103°19′E, 26°35′N); from 2041 to 2060, it is in Liangshan Yi Autonomous Prefecture, Sichuan Province (102°6′E, 26°13′N); from 2061 to 2080, it is in Panzhihua City, Sichuan Province (101°38′E, 26°33′N); and from 2081 to 2100, it is in Lijiang City, Yunnan Province (100°57′E, 26°45′N), with its distribution center moving westward.

## 4 Discussion

By identifying key environmental drivers and projecting future habitat shifts, this study provides spatially explicit guidance for prioritizing *in-situ* conservation zones, establishing ecological corridors, selecting suitable sites for *ex-situ* germplasm banks and assisted migration trials, and thus supports the formulation of climate-smart conservation and cultivation strategies for these medicinal species.

### 4.1 Reliability of the MaxEnt model

Compared with other niche models, the MaxEnt model demonstrates higher accuracy and stability of prediction results when the amount of species sample data is insufficient and the correlation of environmental variables in the distribution area is uncertain (Elith et al., 2006, 2011), and it is widely used in studies on the potential suitable habitats of invasive species (Zeng et al., 2024), endangered species (Hui et al., 2023), and other taxa; the reliability of niche model predictions depends on three key factors: the selection of the niche model, the size and coverage of the sample size, and the type and quantity of environmental factors (Ye et al., 2021b). The core advantage of the MaxEnt model lies in its ability to maximize the use of limited data on species distribution point (Ye et al., 2021a); even in scenarios with scarce samples or significant spatial bias, it can still output reliable habitat assessment results (Gogol-Prokurat, 2011), and the gradient difference in the logistic output format accurately matches the actual changes in habitat suitability (Wang et al., 2017), enhancing the interpretability of the results. In terms of optimization strategies, target group background sampling is more suitable for ecological scenarios than random background sampling, and although the latter significantly reduces computation time by relying on R-language parallel computing, it does not affect model accuracy; finally, through targeted parameter tuning of regularization strength and feature combinations—such as optimizing the MaxEnt model’s regularization multiplier and feature combination parameters by using the ENMTools, tool-overfitting is reduced, and high-precision prediction results are generated, which relatively accurately reflect the response of species to environmental factors (Zhu and Qiao, 2016). When the MaxEnt model simulates the niche of a species, the more comprehensive the niche-related factors used, the higher the simulation accuracy (Zhou et al., 2022), and in this study, the AUC values of the three *Gentiana* species were all greater than 0.9, indicating high prediction reliability.

### 4.2 Dominant environmental factors affecting the distribution of *Gentiana* plants: mechanisms and biological meaning

The spatial distribution of *Gentiana* species is constrained by a suite of environmental factors, with climatic variables being the primary drivers (Thuiller, 2007). However, the high contribution rates of specific variables (e.g., Bio12, Bio6, Bio4) revealed by our MaxEnt model necessitate a deeper ecological and physiological interpretation to understand how and why they intervene in shaping distribution patterns.

The significant dependence of *G. rhodantha* and *G. cephalantha* on annual precipitation (contributing 39.1% and 33.3%, respectively) underscores their adaptation to humid environments. Adequate yearly rainfall ensures sustained soil moisture, which is critical for root hydration, nutrient uptake, and the biosynthesis of medicinal secondary metabolites, a process highly dependent on plant water status (Li et al., 2015). Conversely, *G. rigescens* showed high sensitivity to precipitation of the driest month (Bio14, 11% contribution). This suggests that while tolerant of seasonal variation, its establishment and survival, particularly during seedling recruitment, are vulnerable to acute drought stress. This aligns with observations that soil moisture deficit during key phenological phases leads to significant decline in species richness in alpine meadows (Zhang et al., 2018). The dominance of Bio6 for *G. rhodantha* (36.4% contribution) points to a critical low-temperature threshold for overwintering. Extreme winter minima likely cause cellular frost damage or failure to maintain dormancy, explaining its limited distribution in high-latitude regions (Shen et al., 2017). For *G. cephalantha* and *G. rigescens*, temperature seasonality (Bio4) was paramount (27.2% and 38.6% contribution). These species likely rely on distinct seasonal temperature variations as phenological cues for key life-cycle events such as vernalization, dormancy break, and synchronized flowering. A reduction in seasonal amplitude under climate warming could desynchronize these processes, disrupting reproduction (Memmott et al., 2007). Altitude, a significant factor for all three species, integrates changes in temperature, precipitation, solar radiation, and soil properties. The affinity of Gentiana for higher elevations reflects a classic “cool-moist” alpine niche (Tu et al., 2013). Higher altitudes provide refuge from lowland heat and interspecific competition, while also promoting the accumulation of UV-protective flavonoids, potentially linked to their medicinal potency (Zhang et al., 2019). The hump-shaped species richness pattern along elevation gradients further supports mid-altitudes as optimal zones with favorable temperatures and heterogeneous microhabitats (Carnicero et al., 2022).

In summary, the key variables identified are not mere statistical correlates but represent fundamental ecological filters: water availability sets hydric boundaries, extreme cold defines survival limits, temperature seasonality regulates life history, and altitude integrates multiple stressors to create a suitable microclimatic envelope. This mechanistic understanding reinforces the reliability of our model projections and highlights the specific vulnerabilities each species faces under climate change.

### 4.3 Impact of climate change on the suitable habitats of *Gentiana* plants

Global warming is accelerating, and extreme weather events are becoming more frequent, leading to a shrinkage in the area of suitable habitats for various species and a trend of migration to higher latitudes or altitudes (Chen et al., 2011); due to inherent physiological constraints, the rate at which species distributions adapt to climate change is often lagging (Hof et al., 2011). Studies using multiple methods have pointed out that climate change will cause an overall reduction in the distribution area of more than 80% of rare and endangered medicinal plant species, and a decrease in species diversity in nearly half of the regions (Brown et al., 2020; Huang et al., 2023), which is consistent with the results of this study showing that the suitable habitat area of most *Gentiana* species shrinks under different scenarios and periods; at the same time, some predictions have found that global warming may lead to an increase in the suitable habitat area of *Lonicera japonica*, *Belamcanda chinensis*, and *Tetradium ruticarpum* (Cui, 2015), which is consistent with the conclusion of this study that the suitable habitat area of *Gentiana* cephalantha increases in 2061–2080 and 2081–2100 under the SSP585 scenario; plant species with narrow distribution ranges usually exhibit limited ecological plasticity and are therefore more vulnerable to climate change than widely distributed groups (Ma and Sun, 2018). It is speculated that due to climate warming, plants will migrate to higher altitudes and latitudes (Thomas et al., 2022; Su et al., 2024a, 2024); when predicting the migration of suitable habitats of *Gentiana* plants in this study, it was found that with the rise in global temperatures, the suitable habitats also migrate to high-latitude areas in the north, which may be because the survival areas in low latitudes have exceeded the heat tolerance of the species as temperatures rise, and this is consistent with the research result that migrating to higher altitudes is one of the main strategies for many species to cope with climate change (Zhang et al., 2019; Zu et al., 2021); in addition, under the three climate scenarios, the centroid longitude of the three *Gentiana* species all moves westward, which may be because when the distribution area of plants migrates to higher altitudes, their longitude shifts westward (Liang et al., 2018). Our projections indicate an overall reduction in suitable habitat area for the studied Gentiana species. This net loss, however, reflects a process of spatial restructuring, in which habitat loss in current core areas exceeded the typically fragmented gains observed at higher latitudes and elevations. This outcome contrasts with a coherent, large-scale geographic shift. The observed pattern aligns with the broader biogeographical expectation of species moving toward higher latitudes and altitudes under warming climates (Gu et al., 2021), a trend also documented in China’s forest vegetation (Yang et al., 2015; Wu et al., 2016). These findings underscore the urgency of studying fine−scale habitat dynamics to mitigate climate−change impacts on species distributions.

### 4.4 Limitations and future research directions

Although this study provides novel insights into the climate−driven distribution changes of three key *Gentiana* species, several limitations should be acknowledged. First, our analysis focused on *G. rhodantha*, *G. cephalantha*, and *G. rigescens*—species chosen for their ecological gradient coverage, medicinal value, data availability, and contrasting climatic responses. While this selection enables a robust and representative modeling exercise, it does not capture the full diversity of the genus, which comprises over 200 species in China. Future work should extend similar modeling approaches to other Gentiana species as more occurrence data become available. Second, the MaxEnt model relies primarily on climatic and topographic variables; additional factors such as soil properties, biotic interactions, and human disturbance were not incorporated, which may affect fine−scale habitat suitability. Third, the projections assume that species can disperse freely to newly suitable areas, while in reality dispersal limitations and habitat fragmentation may constrain range shifts. We recommend that subsequent studies integrate dispersal models and land−use data to refine predictions. Finally, the conservation strategies proposed here are based on the responses of three representative species; a comprehensive genus−wide conservation plan will require systematic assessments of all threatened *Gentiana* taxa. Nevertheless, the methodology and findings presented here offer a scalable framework for prioritizing conservation actions and understanding climate−change impacts on medicinal plants in biodiversity hotspots.

### 4.5 Conservation and sustainable management implications

Building upon our findings on habitat shifts and key environmental drivers, we propose a multi-faceted strategy for conservation and sustainable use. First, priority conservation zones should be established within current highly suitable areas (e.g., contiguous regions in Yunnan, Sichuan, and Guizhou), with expanded protection for identified climate refugia at higher elevations along the projected migration paths. Second, for species with severely contracting ranges (e.g., *G. rigescens*), assisted migration trials to future suitable areas and the creation of *ex-situ* germplasm banks from at-risk populations are urgently needed. Third, sustainable harvesting guidelines should be implemented, permitting regulated collection only in stable, high-suitability zones while prohibiting it in areas of projected loss. Furthermore, targeted cultivation can be promoted in optimal regions using the identified climatic parameters (e.g., specific precipitation and temperature ranges) to reduce wild harvesting pressure. Finally, integrating these spatial predictions into national ecological conservation redlines and protected area network planning will ensure policy alignment. This framework enables a proactive, climate-smart approach to safeguarding *Gentiana* resources, balancing ecological integrity with sustainable utilization for traditional medicine.

## 5 Conclusions

This study employed the MaxEnt model to assess the impact of climate change on the potential distribution of three medicinal *Gentiana* species in China. The results project a general reduction in total suitable habitat area under future scenarios, accompanied by a spatial restructuring where habitat losses in current core regions exceed fragmented gains at higher latitudes and elevations. Key environmental drivers were species-specific: annual precipitation (Bio12) and minimum winter temperature (Bio6) primarily shaped *G. rhodantha* distribution, while temperature seasonality (Bio4) and altitude dominated for *G. cephalantha* and *G. rigescens*. To mitigate these impacts, conservation efforts should prioritize *in-situ* protection in current highly suitable areas (e.g., Yunnan, Sichuan, Guizhou), establish *ex-situ* germplasm banks, and plan for assisted migration. These findings provide a scientific basis for the climate-smart conservation and sustainable utilization of these valuable medicinal resources.

## Data Availability

The original contributions presented in the study are included in the article/supplementary material. Further inquiries can be directed to the corresponding author.
